# Curare alkaloids from Matis Dart Poison: Comparison with *d*-tubocurarine in interactions with nicotinic, 5-HT_3_ serotonin and GABA_A_ receptors

**DOI:** 10.1371/journal.pone.0210182

**Published:** 2019-01-04

**Authors:** Ekaterina N. Spirova, Igor A. Ivanov, Igor E. Kasheverov, Denis S. Kudryavtsev, Irina V. Shelukhina, Alexandra I. Garifulina, Lina V. Son, Sarah C. R. Lummis, Gonzalo R. Malca-Garcia, Rainer W. Bussmann, Lothar Hennig, Athanassios Giannis, Victor I. Tsetlin

**Affiliations:** 1 Department of Molecular Neuroimmune signaling, Shemyakin-Ovchinnikov Institute of Bioorganic Chemistry, Russian Academy of Sciences, Moscow, Russia; 2 Sechenov First Moscow State Medical University, Institute of Molecular Medicine, Moscow, Russia; 3 Department of Biochemistry, University of Cambridge, Cambridge, United Kingdom; 4 Department of Medicinal Chemistry and Pharmacognosy, College of Pharmacy, University of Illinois at Chicago, Chicago, IL, United States of America; 5 Museo Nacional de Ciencias Naturales, La Paz, Bolivia; 6 Institut für Organische Chemie, Fakultät für Chemie und Mineralogie, Universität Leipzig, Leipzig, Germany; 7 PhysBio of MEPhI, Moscow, Russia; Weizmann Institute of Science, ISRAEL

## Abstract

Several novel bisbenzylisoquinoline alkaloids (BBIQAs) have recently been isolated from a Matis tribe arrow poison and shown by two-electrode voltage-clamp to inhibit mouse muscle nicotinic acetylcholine receptors (nAChR). Here, using radioligand assay with *Aplysia californica* AChBP and radioiodinated α-bungarotoxin ([^125^I]-αBgt), we show that BBIQA1, BBIQA2, and *d-*tubocurarine (*d*-TC) have similar affinities to nAChR orthosteric site. However, a competition with [^125^I]-αBgt for binding to the *Torpedo californica* muscle-type nAChR revealed that BBIQAs1, 2, and 3 are less potent (IC_50_s = 26.3, 8.75, and 17.0 μM) than *d*-TC (IC_50_ = 0.39 μM), while with α7 nAChR in GH_4_C_1_ cells, BBIQA1 was less potent that *d*-TC (IC_50_s = 162 μM and 7.77 μM, respectively), but BBIQA2 was similar (IC_50_ = 5.52 μM). In inhibiting the Ca^2+^ responses induced by acetylcholine in Neuro2a cells expressing the mouse adult α1β1εδ nAChR or human α7 nAChR, BBIQAs1 and 2 had similar potencies to *d*-TC (IC_50_s in the range 0.75–3.08 μM). Our data suggest that BBIQA1 and BBIQA2 can inhibit adult muscle α1β1εδ nAChR by both competitive and noncompetitive mechanisms. Further experiments on neuronal α3β2, α4β2, and α9α10 nAChRs, expressed in *Xenopus laevis* oocytes, showed that similar potencies for BBIQAs1, 2, and *d*-TC. With α3β2γ2 GABA_A_R currents were almost completely inhibited by *d*-TC at a high (100 μM) concentration, but BBIQAs1 and 2 were less potent (only 40–50% inhibition), whereas in competition with Alexa Fluor 546-α-cobratoxin for binding to α1β3γ2 GABA_A_R in Neuro2a cells, *d*-TC and these analogs had comparable affinities. Especially interesting effects of BBIQAs1 and 2 in comparison with *d*-TC were observed for 5-HT_3A_R: BBIQA1 and BBIQA2 were 5- and 87-fold less potent than *d*-TC (IC_50_ = 22.63 nM). Thus, our results reveal that these BBIQAs differ from *d*-TC in their potencies towards certain Cys-loop receptors, and we suggest that understanding the reasons behind this might be useful for future drug design.

## Introduction

The nicotinic acetylcholine (nAChR), glycine (GlyR), serotonin (5-HT_3_R), and γ-aminobutyric acid (GABA_A_R) receptors belong to the Cys-loop family of ligand-gated ion channels (LGICs) [1–5]. All members of this family have a similar structure: five subunits form a pentamer surrounding a selective ion channel; for nAChR and 5-HT_3_R, the ion channel is permeable to cations, while in GABA_A_R and GlyR it is anion-permeable. Each subunit is composed of an N-terminal extracellular domain, four transmembrane fragments (M1-M4), and a large cytoplasmic loop between M3 and M4 fragments. In the N-terminal extracellular domain, there is a highly conserved 13 amino acid loop tethered at each end by a cysteine disulfide bridge, hence the name “Cys-loop receptors” [6–8].

Ligands for Cys-loop receptors are very diverse and include both synthetic and natural compounds. Typically, agonists and competitive antagonists bind to the orthosteric sites located between two adjacent subunits, whereas allosteric binders target different sites [9–11]. Cys-loop receptors are widely expressed in the central and peripheral nervous systems, where they mediate fast synaptic neurotransmission, and also in other systems, for example, in immune and epithelial cells [12–16]. Drugs that act at Cys-loop receptors are used as muscle relaxants, chronic pain therapeutics, anti-emetics, neurodegenerative and psychiatric therapeutics, smoking cessation aids and new compounds are being actively sought [17–20]. Muscle relaxants and/or neuromuscular blocking agents are widely used in anesthesia, where they act by interrupting transmission at the neuromuscular junction [21]. The development of clinical muscle relaxants began with the examination of the arrow poison produced from plant *Chondodenron tomentosum* by South American Indians [21, 22] and by isolation of *d*-tubocurarine (*d*-TC) [23]. *d*-TC is an antagonist of the end-plate muscle type nAChRs, which are composed by two α1, one β1, one δ, and either γ (fetal) or ɛ (adult) subunits. The orthosteric *α*/ε (or *α*/*γ)* site binds *d*-TC with 100-fold higher affinity than the orthosteric *α*/*δ* site [24]. In the middle of the last century, *d*-TC was a popular choice in surgery, although there were some adverse effects on blood pressure and the cardiovascular system [22, 25]. Today the compounds used are much safer, but still so there are some side effects and there is a strong need in understanding the key structural elements important for high selectivity of the muscle relaxants. In particular, in addition to high selectivity, the perfect relaxant should be characterized by rapid onset and short or intermediate duration of action [21, 26, 27].

**Fig 1:**
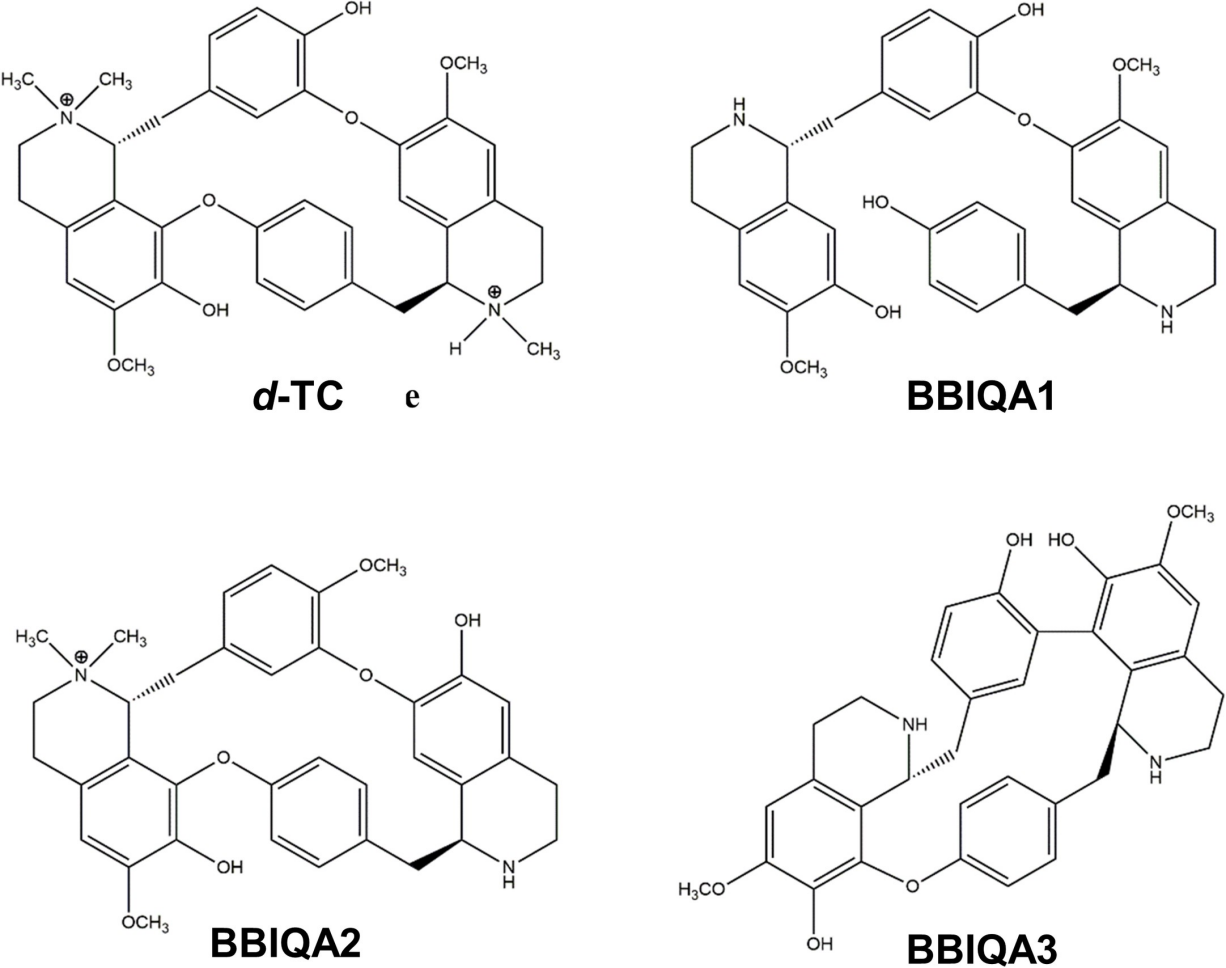
Chemical structures of *d*-tubocurarine (*d*-TC) and bisbenzyltetrahydroisoquinoline alkaloids (BBIQAs).

We have recently described an analysis of a poison which the Matis tribe in South America is using for anointing their arrows [28]. In addition to a number of well-known compounds, such as magniflorine, lindoldhamine and some others [29, 30], a series of novel bisbenzyltetrahydroisoquinoline alkaloids (BBIQAs) (Fig 1) were discovered. Due to their structural similarity with *d*-TC, it was reasonable to expect from them the inhibition of neuromuscular transmission. Indeed, it was found that both the crude venom (at a concentration of 1.0 mg/mL), and individual BBIQAs at the relatively high concentration of 20 μM, efficiently decreased the amplitudes of ACh-induced currents in mouse muscle nAChR heterologously expressed in *X*. *laevis* oocytes [28].

The aim of the present work was a detailed *in vitro* comparison of *d*-TC and BBIQAs activities on a set of muscle-type and neuronal nAChRs. Previous work has shown that *d*-TC is able to block nAChRs competitively and noncompetitively, depending on the nAChR subtype: inhibition of neuronal α4β2 nAChR is competitive, while muscle-type and neuronal α3β4 nAChRs are blocked by a mixed mechanism involving binding to both orthosteric and allosteric sites [31]. *d*-TC binds with a high affinity to acetylcholine-binding protein (AChBP), a good model for studying agonist and competitive antagonist actions on nAChR [32]. Interestingly, *d*-TC adopts three different orientations in the AChBP ligand binding sites [33], providing a possible reason for the wide range of receptors targeted by *d*-TC: in addition to nAChRs, *d*-TC is an effective blocker of 5-HT_3_R (with IC_50_ values of 11.4–13 nM and 1.8 μM for mouse and human 5-HT_3A_R, respectively [34, 35]), and at higher concentrations (30 μM) it also inhibits GABA_A_R [36]. In the current research we compared the effects of *d*-TC and the novel BBIQAs on muscle-type and neuronal nAChRs, as well as on 5-HT_3A_R and GABA_A_R. Our data suggest that BBIQAs do not completely mimic the effects of *d*-TC and thus could help in the identification of structural motifs responsible for selectivity of distinct compounds, which is valuable for future drug design.

## Materials and methods

### Materials

The Matis poison (5.29 g) was obtained in Leticia, at the Colombian-Brazilian border, by Dr. Rainer W. Bussmann. The material was authenticated and deposited at the William L. Brown Center, Missouri Botanical Garden, in November 2011. Powdered material was maintained at room temperature (22–25°C) and protected from light until required for extraction and analysis. *T*. *californica* electric organ membranes were a generous gift from Ferdinand Hucho (Free University of Berlin, Berlin, Germany) and GH_4_C_1_ cells–from EliLilly (London, UK). Mouse neuroblastoma Neuro2a cells were purchased from the Russian collection of cell cultures (Institute of Cytology, Russian Academy of Sciences, Saint Petersburg, Russia). Mature *X*. *laevis* female frogs used in this study were obtained commercially (NASCO, Fort Atkinson WI, USA) and housed in a facility with 12:12 hours light:dark cycles, 18–20°C ambient temperature. Animals were fed twice a week and maintained according to supplier recommendations (https://www.enasco.com/page/xen_care). This study was carried out in strict accordance with the World Health Organization’s International Guiding Principles for Biomedical Research Involving Animals. The protocol was approved by the Institutional Policy on the Use of Laboratory Animals of the Shemyakin-Ovchinnikov Institute of Bioorganic Chemistry RAS (Protocol Number: 251/2018 26.02.18). All frog surgery was performed under benzocaine anesthesia, and all the appropriate actions were taken to minimize discomfort to frogs.

### Samples purification

Preparative purification was carried out with a Gilson HPLC system (322 pump with GX 271 liquid handler) equipped with an RP column and 155 UV-Vis detector, set at 210 and 254 nm. Compounds were eluted with a H_2_O-MeCN gradient with 0.1% CF_3_COOH. UPLC-MS analysis was performed using Thermo Finnigan LCQ Deca XP Plus ion trap instrument with Thermo Accela UPLC system equipped with YMC Triart column (C-18 150 × 2 mm, 1.9 μm). Detection was achieved by UV-VIS DAD (190–600 nm) and full scan MS (ESI+, 150–2000 au). Crude samples were dissolved in mixture of water/methanol/acetic acid 88:10:2 to a final concentration of 3 mg/mL, filtered through a 45 μm nylon filter and injected into the LC system. Chromatography was carried out using a Phenomenex Luna C18(2) 5 μm 21.2x150 mm column in a linear gradient from 9 to 55% of acetonitrile in 15 Column Volumes. Fractions of interest were collected, analyzed by UPLC-MS and lyophilized.

### Computer modeling with *A*. *californica* AChBP

Docking experiments were performed using Autodock 4.2 and analyzed with MGL Tools 1.5.6 [37] with parameters set as follows: genetic algorithm population size 150, number of evaluations 25000000 and number of runs 100. The structure of *A*. *californica* AChBP co-crystallized with *d*-TC was used as a receptor (PDB 2XYT) which contains *d*-TC in three distinct binding positions [33]. To address the possibility of different binding modes we performed docking of BBIQA1 and BBIQA2 to all three types of binding sites. Results were clustered and inspected using MGL Tools 1.5.6 and visualized using UCSF Chimera [38].

### Radioligand assay

In competition experiments with [^125^I]-αBgt, the compounds were pre-incubated 3 h at room temperature with *A*. *californica* AChBP at final concentrations of 140 nM, *T*. *californica* electric organ membranes (final concentration 1.25 nM of toxin-binding sites) or GH_4_C_1_ cells (6.5 μg of total protein with final concentration of 0.4 nM of toxin-binding sites) in 50 μL of binding buffer (20 mM Tris-HCl buffer, 1 mg/mL of bovine serum albumin, pH 8.0). [^125^I]-αBgt was then added to cells or membranes to a final concentration of 0.1–0.2 nM and the mixtures were incubated for 5 min. Binding was stopped by rapid filtration on GF/C filters (Whatman, UK) pre-soaked in 0.25% polyethylenimine, unbound radioactivity being removed from the filters by washing (3×3 mL) with binding buffer. Non-specific binding was determined in all cases using a 3 h pre-incubation with 30 μM α-cobratoxin (αCtx). The results were analyzed using OriginPro 2017 (OriginLab Corporation, Northampton, MA, USA) fitting to a dose-response curve with a variable Hill slope using the equation y = A1 + (A2-A1)/(1 + 10^(LOGx0-x)*p^): where A1 and A2 are bottom and top asymptotes, respectively; p–Hill slope; LOGx0 –log_10_[IC_50_, M].

### Cell culturing and transfection

Mouse neuroblastoma Neuro2a cells were cultured in Dulbecco’s modified Eagle’s medium (DMEM, Paneco, Russia) supplemented with 10% FBS (PAA Laboratories, Austria). Cells were sub-cultured 24 h before transfection and plated at a density of 10,000 cells per well (black 96-well plate, Corning, USA), followed by lipofectamine (Invitrogen, USA)-mediated transient co-transfection of human α7 nAChR-pCEP4, fluorescent calcium sensor pCase12-cyto (Evrogen, Russia) and chaperone Ric3-pCMV6-XL5 or NACHO TMEM35-pCMV6-XL5 plasmid constructs (OriGene, USA). Mouse muscle α1, β1, ε, and δ nAChR-pRBG4 plasmid constructs were expressed similarly, but without a chaperone, as well as mouse α1, β3, γ2 GABA_A_R-lab-pCI plasmids.

### Calcium imaging

Calcium imaging was performed as described previously [39]. Briefly, cell medium was removed and cells were washed with external buffer containing (in mM) 140 NaCl, 2 CaCl_2_, 2.8 KCl, 4 MgCl_2_, 20 HEPES, 10 glucose at pH 7.4. Cells were pre-incubated with *d*-TC, BBIQA1, or BBIQA2 for 20 min at room temperature, or with αBgt for 5 min at room temperature. To potentiate α7 nAChR responses, PNU120596 (10 μM) was added to the pre-incubation solution. Cells were excited at 485 nm and emitted fluorescence was detected at 535±10 nm, using a multimodal microplate reader Hidex Sense (Hidex, Turku, Finland). Fluorescence was recorded every 2 s for 3 min following agonist addition. Responses were measured as peak intensity minus basal fluorescence level, and are expressed as a percentage of a maximal response to agonist. Negative controls were run in the presence of 5 μM αCtx. Data files were analyzed using HidexSence software (Hidex, Turku, Finland) and then the results were analyzed using OriginPro 2017 (OriginLab Corporation, Northampton, MA, USA) fitting to a dose-response curve with a variable Hill slope using the equation y = A1 + (A2-A1)/(1 + 10^(LOGx0-x)*p^): A1 and A2 are bottom and top asymptotes, respectively; p–Hill slope; LOGx0 –log_10_[IC_50_ or EC_50_, M].

### Fluorescence assay

Fluorescence assays were performed as previously described [40]. Neuro2a cells transiently expressing α1β3γ2 GABA_A_R were washed with the external buffer. Cells were then pre-incubated with 50 μM *d*-TC, BBIQA1, or BBIQA2 for 15 min at room temperature followed by 20 min incubation with 50 nM Alexa Fluor 546 αCtx conjugate in a final volume of 100 μL. Cells were then washed 3 times with two-fold excess buffer. Non-specific fluorescence was determined using 3 μM αCtx. Pictures of 3 independent fields chosen in blind mode in each plate well were taken using an epifluorescent microscope IX71 (Olympus, Japan) equipped with a digital CCD camera. Fluorescence intensity was counted using CellX and ImageJ open-source software. Fluorescence intensity was normalized to the mean integral intensity of the field incubated in presence of 50 nM Alexa Fluor 546 αCtx conjugate. Each experimental point is an average of integral intensity independently measured for 12 independent fields.

### Electrophysiology

Plasmid pT7TS constructs of human nAChR α3, α9, α10, and β2 subunits were linearized with Xba I (NEB, USA), and the plasmid pGEMHE construct with the mouse 5-HT_3A_ subunit–with Nhe I (NEB, USA). Linearized plasmid constructs were subjected to *in vitro* cRNA transcription using the T7 mMessage mMachine transcription kit (AMBION, USA). Stage V-VI *X*. *laevis* oocytes were defolliculated with 2 mg/mL collagenase Type I (Life Technologies, USA) at room temperature (21–24°C) for 2 h in Ca^2+^-free Barth’s solution composed of (in mM) 88 NaCl, 1.1 KCl, 2.4 NaHCO_3_, 0.8 MgSO_4_, and 15 HEPES-NaOH at pH 7.6. Oocytes were injected with 9.2 ng of cRNAs of human nAChR α3β2, α9α10 (in a ratio 1:1), or mouse 5-HT_3A_R, or 2–3 ng of cDNAs of rat α4β2 (in a ratio 1:1, pcDNA3.1 vector), or mouse α3, β2, and γ2 GABA_A_R subunits in pCI vector. Oocytes were incubated at 18ºC in regular Barth’s solution composed of (in mM) 88 NaCl, 1.1 KCl, 2.4 NaHCO_3_, 0.3 Ca(NO_3_)_2_, 0.4 CaCl_2_, 0.8 MgSO_4_, and 15 HEPES-NaOH at pH 7.6, supplemented with 40 μg/mL gentamicin and 100 μg/mL ampicillin for 4 days before electrophysiological recordings. Two-electrode voltage clamp recordings were made using a turbo TEC-03X amplifier (Npi electronic, Germany) and WinWCP recording software (University of Strathclyde, UK), at a holding potential of -60 mV. Oocytes were briefly washed with Barth’s solution (for human nAChR α3β2, rat nAChR α4β2, mouse 5-HT_3A_R, and mouse α3β2γ2 GABA_A_R) or Ba^2+^ Ringer’s solution composed of (in mM) 115 NaCl, 2.5 KCl, 1.8 BaCl_2_, 10 HEPES at pH 7.2 (for human α9α10 nAChR) followed by 3 applications of agonist. Washout with Barth’s or Ba^2+^ Ringer’s solution was done for 5 min between agonist applications. Oocytes were pre-incubated with *d*-TC, BBIQA1, or BBIQA2 for 5 min followed by co-application with agonist (3 s). Peak current amplitudes of agonist-evoked responses were measured before and after pre-incubation of oocytes with *d*-TC, BBIQA1, or BBIQA2. The ratio between these two measurements was used to assess the activity of *d*-TC, BBIQA1, or BBIQA2 on human nAChR α3β2, α9α10, rat α4β2, mouse 5-HT_3A_R, and mouse α3β2γ2 GABA_A_R. The results for 5-HT_3A_R were analyzed using OriginPro 2017 (OriginLab Corporation, Northampton, MA, USA) fitting to a dose-response curve with a variable Hill slope using the equation y = A1 + (A2-A1)/(1 + 10^(LOGx0-x)*p^): A1 and A2 are bottom and top asymptotes, respectively; p–Hill slope; LOGx0 –log_10_[IC_50_, M].

### Data and statistical analysis

Data are presented as mean with 95% confidence interval (CI) or mean ± SEM for the indicated number of independent experiments (n). Statistical analysis (One-way ANOVA with Tukey’s HSD test) was performed using OriginPro 2017 software (OriginLab Corporation, Northampton, MA, USA). In all the tests, *p <* 0.05 was taken as significant.

## Results

### Computer modeling with *A*. *californica* AChBP

Molecular docking studies were performed to detect possible activity against nAChRs. It was not unexpected for BBIQAs to bind to nAChR because they have structures similar to that of *d*-TC. However, such intuitive suggestion should be tested with *A*. *californica* AChBP, co-crystallized with the *d*-TC, an excellent nAChR model for this work [33]. Docking results showed the potential for high affinity binding of BBIQA1 and BBIQA2 at the *A*. *californica* AChBP binding site (S1 Table). BBIQA1 docking poses differed substantially from the experimentally observed *d*-TC orientations, possibly due to greater structure flexibility (S1A Fig), while predicted BBIQA2 binding poses (S1B Fig) were similar. All docking simulations predicted BBIQA1 (S1C Fig) and BBIQA2 (S1D Fig) to bind at the classic orthosteric site under loop C of AChBP.

### Samples purification

All experiments were carried out on BBIQAs1-3 after additional HPLC purification and using individual peaks according to analytical HPLC (S2–S7 Figs). The mass spectra given for each of these compounds provide further confirmation of their purity and structure.

### Radioligand Assay

Radioligand analysis was performed using [^125^I]-α-bungarotoxin (αBgt) with a specific activity of 500 Ci/mmol and either *A*. *californica* AChBP, membrane-bound nAChRs from the electric organ of the *T*. *californica* ray, or human α7 nAChR in GH_4_C_1_ cells as previously described [41].

**Fig 2:**
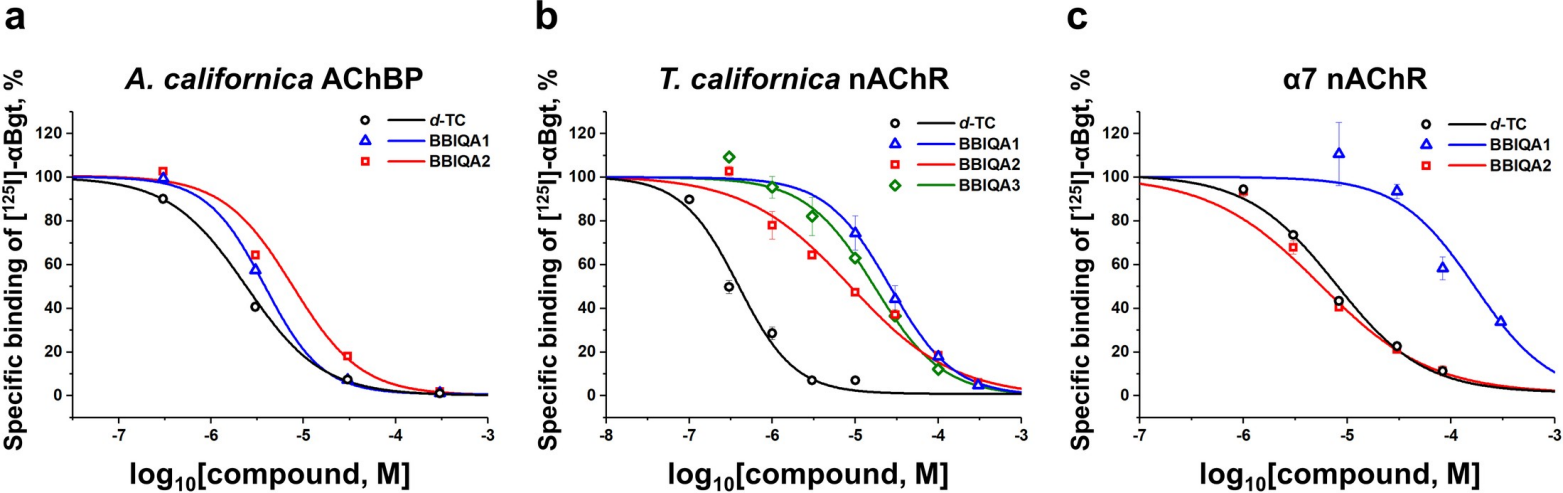
Inhibition of [^125^I]-labeled α-bungarotoxin binding. (a) to *A*. *californica* AChBP, (b) to nAChR from *Torpedo californica* electric organ membranes, and (c) to human α7 nAChR in GH_4_C_1_ cells with BBIQA1 (*open blue triangles*), BBIQA2 (*open red squares*), BBIQA3 (*open green rhombuses*), and *d*-TC (*open black circles*). Data are mean ± SEM of two biological replicates with duplicates for each point (the number of technical replicates is 2), i.e. n = 4. IC_50_ values derived from these data are shown in Table 1.

BBIQA1 and BBIQA2 demonstrate a little lower ability to compete with [^125^I]-αBgt for binding to *A*. *californica* AChBP in comparison with *d*-TC, their IC_50_ values were 3.80 μM, 7.63 μM, and 2.41 μM, respectively (Fig 2A and Table 1). In case of *T*. *californica* nAChR (Fig 2B and Table 1) the lowest affinity was registered for BBIQA1 (IC_50_ 26.3 μM), with slightly higher values for BBIQA3 (17 μM), and BBIQA2 (8.75 μM). However, even BBIQA2 was 22-fold less potent than *d-TC* (IC_50_ 0.39 μM). Inhibition of specific [^125^I]-αBgt binding to α7 nAChR (Fig 2C) revealed that the affinity of BBIQA1 is low (IC_50_ 162 μM), but that of BBIQA2 is similar to *d*-TC (IC_50_ 5.52 μM and 7.77 μM, respectively).

**Table 1:** Inhibitory effects of the alkaloids from Matis Dart Poison and *d*-TC on [^125^I]-αBgt binding, agonist-induced calcium responses, and/or currents mediated by *A*. *californica* AChBP, *T*. *californica* nAChR, mouse adult α1β1εδ nAChR, human α7 nAChR, and mouse 5-HT_3A_R.

Compound	***Radioligand assay***					
	***A*. *californica* AChBP**		***T*. *californica* nAChR**		**α7 nAChR**	
	**IC**_**50**_, μM	nH	**IC**_**50**_, μM	nH	**IC**_**50**_, μM	nH
	(95% CI)	(mean ± SEM)	(95% CI)	(mean ± SEM)	(95% CI)	(mean ± SEM)
***d*-TC**	**2.41**	-1.05 ± 0.02	**0.39**	-1.28 ± 0.11	**7.77**	-1.02 ± 0.05
	(2.26–2.56)		(0.31–0.47)		(6.72–8.81)	
**BBIQA1**	**3.80**	-1.39 ± 0.09	**26.3**	-1.13 ± 0.02	**162**	-1.19 ± 0.10
	(3.47–4.13)		(24.7–27.8)		(148–191)	
**BBIQA2**	**7.63**	-1.16 ± 0.11	**8.75**	-0.69 ± 0.05	**5.52**	-0.82 ± 0.05
	(5.28–9.97)		(7.22–10.27)		(4.50–6.55)	
**BBIQA3**	−	−	**17.0**	-1.05 ± 0.02	−	−
			(16.4–17.7)			
Compound	***Calcium Imaging***				***Electrophysiology***	
	**α1β1εδ nAChR**		**α7 nAChR**		**5-HT**_**3A**_**R**	
	**IC**_**50**_, μM	nH	**IC**_**50**_, μM	nH	**IC**_**50**_, nM	nH
	(95% CI)	(mean ± SEM)	(95% CI)	(mean ± SEM)	(95% CI)	(mean ± SEM)
***d*-TC**	**0.81**	-1.87 ± 0.41	**2.03**	-1.39 ± 0.21	**22.63**	-1.19 ± 0.21
	(0.50–1.12)		(1.31–2.76)		(10.49–34.78)	
**BBIQA1**	**0.75**	-0.96 ± 0.16	**1.70**	-0.83 ± 0.17	**119.4**	-1.06 ± 0.09
	(0.28–1.21)		(1.11–2.61)		(74.4–164.4)	
**BBIQA2**	**1.75**	-1.04 ± 0.21	**3.08**	-0.94 ± 0.29	**1975**	-1.83 ± 0.01
	(0.88–2.61)		(1.47–4.68)		(1861–2090)	

### Calcium imaging

**Fig 3:**
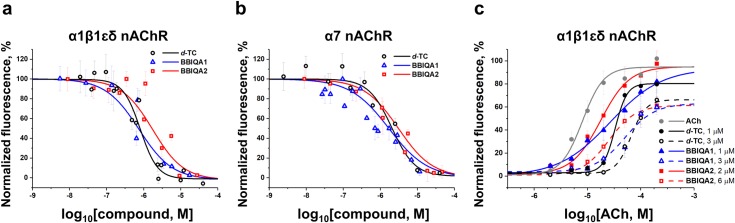
Dose-response curves of inhibitory activity of *d*-TC (*open black circles*), BBIQA1 (*open blue triangles*), and BBIQA2 (*open red squares*). (a) on the 30 μM (approx. EC_50_) acetylcholine-evoked intracellular calcium ion concentration ([Ca^2+^]_i_) rise in Neuro2a cells expressing mouse adult α1β1εδ nAChRs; and (b) on the 10 μM (approx. EC_50_) acetylcholine-evoked [Ca^2+^]_i_ rise in Neuro2a cells expressing human α7 nAChRs in the presence of 10 μM PNU120596. Data are presented as mean ± SEM, n = 3. The respective IC_50_ values are shown in the Table 1. (c) Dose-response curves of acetylcholine (ACh)-evoked [Ca^2+^]_i_ rise in the absence (*grey circles*, EC_50_ = 8.4 ± 0.6 μM) and presence of *d*-TC (*black circles*) or its analogs, BBIQA1 (*blue triangles*), and BBIQA2 (*red squares*) at different concentrations in Neuro2a cells expressing mouse adult α1β1εδ nAChRs. Data are presented as mean ± SEM, n = 3. EC_50_ values are shown in the Table 2.

The next step was the analysis of BBIQAs interactions with mouse adult muscle nAChR (α1β1εδ) (Fig 3A and 3C) which differs by one subunit (ε) from the *T*. *californica* nAChR (α1β1γδ). This receptor was heterologously expressed in Neuro2a cells and the inhibitory effects of BBIQAs on the ACh-induced increase in intracellular Сa^2+^ concentration ([Ca^2+^]_i_) were monitored based on the fluorescence of the co-expressed calcium ion sensor Case12 as described in [39]. As seen from Fig 3A and Table 1, BBIQA1 and *d*-TC have virtually identical IC_50_ values (0.75 and 0.81 μM, respectively) and BBIQA2 is ~2-fold less potent (1.75 μM). The same relationship was observed for the human α7 nAChR expressed in Neuro2a cells and analyzed in the presence of a positive allosteric modulator PNU120596 (Fig 3B). Due to extremely rapid desensitization rate of α7 nAChR, its kinetics is too fast to be measured using any conventional calcium imaging techniques [42–45]. Instead, the application of its positive allosteric modulator PNU120596 significantly decreases α7 nAChR desensitization rate providing the possibility to monitor as the receptor-mediated intracellular calcium raises [46, 47]. PNU120596 binds to the transmembrane domain of the α7 receptor leading to an increase of the maximal agonist-elicited response, agonist potency and apparent cooperativity [48–50]. In general, positive allosteric modulators have little to no effect on equilibrium binding of α7 competitive antagonists, because these modulators bind in a non-competitive manner, away from the traditional agonist binding site [51]. However, we must be cautious with calcium imaging data interpretation, as PNU120596 could affect the data on inhibition of α7 nAChR by BBIQAs due to their complex character of interaction.

The IC_50_ values for *d*-TC and BBIQA1 were 2.03 and 1.70 μM, respectively, while for BBIQA2 this value was 3.08 μM. Data on the inhibitory activity of BBIQAs from the calcium imaging experiments and from the above-described data on the muscle-type and α7 nAChRs obtained in radioligand experiments are shown in Table 1.

To determine possible differences between *d*-TC and its BBIQA1 / BBIQA2 analogs in binding to orthosteric or allosteric sites, we performed a series of calcium imaging experiments with Neuro2a cells expressing mouse adult α1β1εδ nAChR (Fig 3C). *d*-TC or its analogs at different concentrations (at IC_50_ and 3x IC_50_, see Table 1: Calcium Imaging for α1β1εδ nAChR) were co-applied along with acetylcholine to Neuro2a cells expressing α1β1εδ nAChR. As shown in Fig 3C, the presence of *d*-TC, BBIQA1, or BBIQA2 shifts the acetylcholine dose response curve to the right, with an increased acetylcholine EC_50_ value (Table 2), and reduces the maximal acetylcholine response (Fig 3C).

**Table 2:** Inhibitory effects of the alkaloids from Matis Dart Poison and *d*-TC at different concentrations (IC_50_ and 3x IC_50_) on the acetylcholine dose–response curve at mouse adult α1β1εδ nAChR expressed in Neuro2a cells.

Compound	**[Ca**^**2+**^**]**_**i**_ **rise**			
	**α1β1εδ nAChR**			
**ACh**	**EC**_**50**_, μM (95% CI)		**nH** (mean ± SEM)	
	**8.04** (5.04–11.04)		2.19 ± 0.41	
Compound	**IC**_**50**_ **concentration**		**3x IC**_**50**_ **concentration**	
	**EC**_**50**_, μM	**nH**	**EC**_**50**_, μM	**nH**
	(95% CI)	(mean ± SE)	(95% CI)	(mean ± SE)
**ACh + *d*-TC**	**33.05**	3.90 ± 1.28	**65.32**	3.11 ± 0.78
	(17.27–48.82)		(41.28–89.36)	
**ACh + BBIQA1**	**26.53**	0.90 ± 0.05	**50.44**	1.55 ± 0.34
	(19.83–33.23)		(23.90–76.98)	
**ACh + BBIQA2**	**17.63**	1.60 ± 0.34	**25.22**	1.74 ± 0.14
	(8.68–26.58)		(21.15–29.29)	

### Electrophysiology

**Fig 4:**
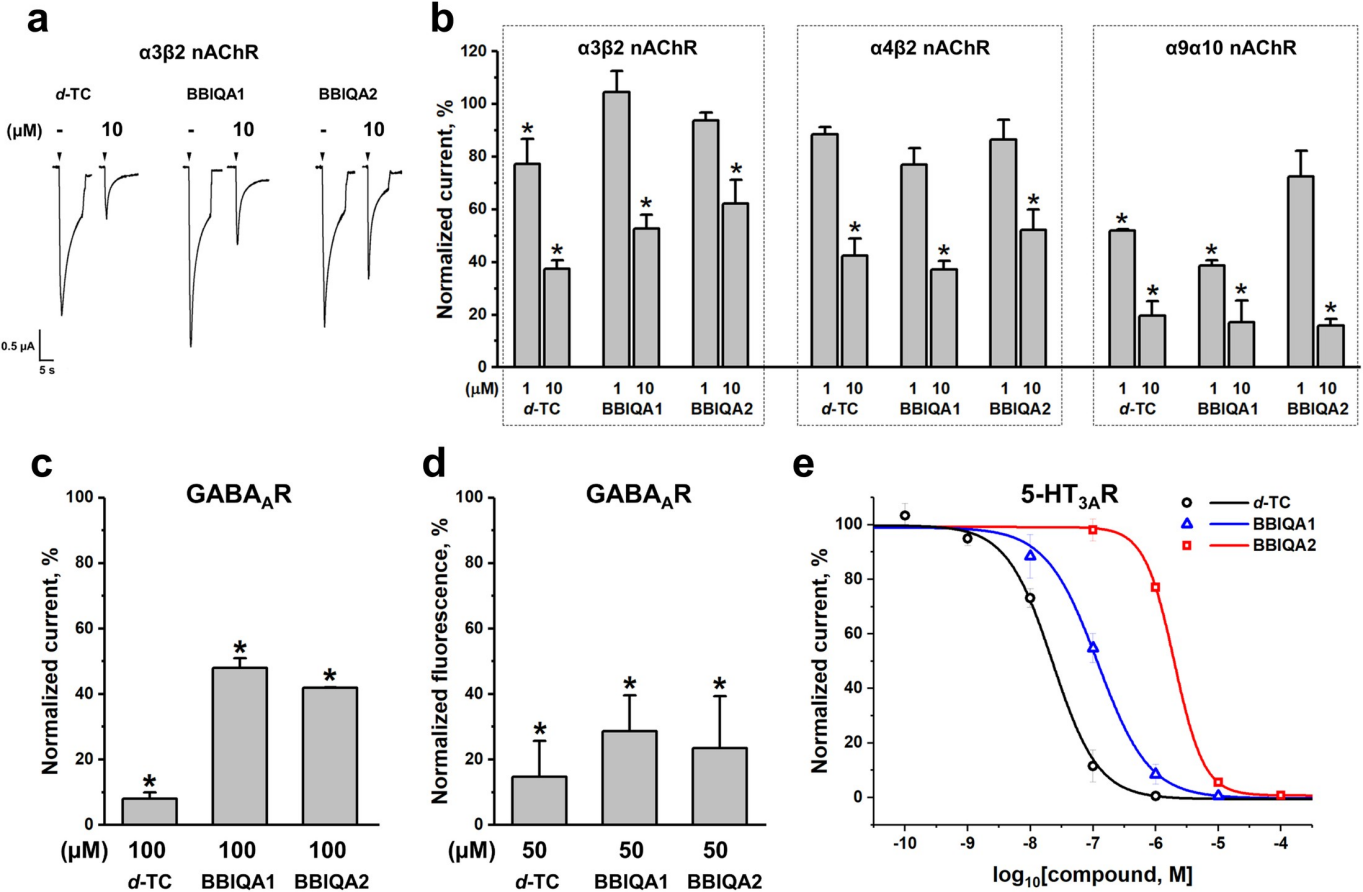
Activity of *d*-TC, BBIQAs1 and 2 against heteromeric neuronal nAChRs, GABA_A_R, and 5-HT_3A_R. (a) Representative current traces of human α3β2 nAChR, showing inhibition of nicotine (50 μM)-induced current by 10 μM *d*-TC, BBIQA1, or BBIQA2. (b) Bar graph for *d*-TC and BBIQAs (1 and 10 μM) inhibition of agonist-evoked currents mediated by human α3β2 (50 μM Nicotine), rat α4β2 (10 μM Nicotine), and human α9α10 (25 μM Acetylcholine) nAChRs. (c) Bar graph for *d*-TC and BBIQAs (100 μM) inhibition of agonist-evoked currents mediated by mouse α3β2γ2 GABA_A_R (100 μM GABA). (d) Inhibition of Alexa Fluor 546 α-cobratoxin (αCtx, 50 nM) binding to α1β3γ2 GABA_A_R expressed in Neuro2a cells by 50 μM *d*-TC, BBIQAs 1 and 2. The bar graph represents the remaining fluorescence of Alexa Fluor 546 αCtx (50 nM). In (a, b, c, d) data are presented as mean ± SEM, n = 3–6. One-way ANOVA with Tukey’s HSD test, (*black asterisks*, *p*<0.05, normalized current evoked by agonist in the presence of *d*-TC, BBIQA1, or BBIQA2 vs normalized current induced by agonist in the absence of antagonists). (e) Dose-response curves of *d*-TC (*open black circles*), BBIQA1 (*open blue triangles*), or BBIQA2 (*open red squares*) inhibitory action on 1 μM 5-HT-evoked ion currents mediated by mouse 5-HT_3A_R. Data are presented as mean ± SEM, n = 3–5. IC_50_ values determined from these data are shown in Table 1.

We also analyzed the interactions of *d*-TC, BBIQAs1 and 2 with several subtypes of heteromeric neuronal nAChRs, namely α3β2, α4β2, and α9α10, following their expression in *X*. *laevis* oocytes. As is seen in Fig 4A and 4B, both BBIQAs and *d*-TC have similar inhibitory activities against all three neuronal nAChR subtypes. We also wanted to check whether *d*-TC and BBIQAs differ in their activity towards other Cys-loop receptors. Fig 4C shows that a strong (90%) inhibition of the mouse α3β2γ2 GABA_A_R is observed with a high concentration of *d*-TC (100 μM), while BBIQAs1 and 2 at this concentration produce only a 40–50% inhibition.

As *d*-TC is known to potently inhibit 5-HT_3А_R [34, 35], we checked whether such a property is also inherent in BBIQAs (Fig 4E). A considerable difference was detected between BBIQAs1 and 2: the IC_50_ value for the former was 119.4 nM, while for the latter was 1975 nM, and these two compounds were 5- and 87-fold less active than *d*-TC, the IC_50_ value of which is 22.63 nM (IC_50_ values and 95% confidence intervals are shown in Table 1).

### Fluorescence assay

For the GABA_A_R, we compared functional effects of the compounds on GABA-induced currents with their binding ability: Fig 4D shows that *d*-TC and BBIQAs 1 and 2 have similar potency in competition assays with the fluorescent derivative of αCtx, which may bind to both orthosteric and allosteric sites [40].

## Discussion

In this study we compared the effects of *d*-TC and the structurally-related BBIQAs on the muscle-type and neuronal nAChRs, as well as on 5-HT_3A_R and GABA_A_R. The data obtained suggest that the BBIQAs do not completely mimic the effects of *d*-TC, and thus could provide useful tools for future studies.

Computer modeling with *A*. *californica* AChBP revealed that the BBIQAs may bind to Cys-loop receptors with high affinity at the orthosteric sites, as *d*-TC does. Indeed, radioligand binding assay with *A*. *californica* AChBP showed that both BBIQA1 and BBIQA2 had the affinity almost indistinguishable from that of *d*-TC. However, radioligand assays with *T*. *californica* nAChR revealed that BBIQAs are significantly less potent than *d*-TC in their ability to inhibit binding of [^125^I]-αBgt. It is well-established that αBgt interacts with the binding sites for agonists and competitive antagonists (in other words, binds to the orthosteric sites [52, 53]), so our results suggest that the BBIQAs are less potent than *d*-TC in binding to that site. In calcium imaging experiments on the adult muscle nAChR, the IC_50_ values for BBIQA1 and BBIQA2 were roughly equal to those of *d*-TC, in being in the micromolar range and indicating that *d*-TC, BBIQAs 1 and 2 cannot be considered as highly potent drugs. However, the analysis of the relationship between the neuromuscular blocking dose and duration of action revealed that it is reciprocal, and the rapid onset and short duration of action are associated with drugs of low potency [26, 54, 55]. Therefore, low potency of BBIQAs, as well as of *d*-TC, may be a positive characteristic in terms of their drug-like properties, justifying further structure-functional analysis of these compounds.

Interestingly, our calcium imaging data for muscle α1β1εδ nAChR revealed the BBIQAs potencies similar to that of *d*-TC, in contrast to radioligand assay data where all three BBIQAs were much weaker than *d*-TC, indicating that inhibition of a functional response was more complex than a simple competition. We assumed that these data could be explained by the BBIQAs binding to multiple sites on the receptor: nAChRs are large multisubunit proteins with various binding sites, orthosteric and allosteric, both in the LBD and in the transmembrane domain [56–58]. Indeed, it is well known that *d*-TC is able to target both the orthosteric and allosteric sites [24, 59–62].

Calcium imaging is a convenient method for probing nAChR inhibition [39, 47, 63]. In [47] the researchers used it with genetically encoded calcium ion sensor to study the mechanism of nicotinic receptors (α7 and α4β2 nAChRs) inhibition by well-known competitive antagonists (methyllycaconitine and dihydro-β-erythroidine, respectively). In the presence of competitive antagonist, the agonist dose-response curve shifts to the right with no reduction in the amplitude of the maximal response. Noncompetitive inhibition could as well be detected by calcium imaging with genetically encoded calcium ion sensor Case12. It was previously detected that baptide 2, a short peptide from puff adder Bitis arietans venom, inhibits α1β1εδ nAChR in noncompetitive way: the pre-incubation with baptide 2 doesn’t shift the acetylcholine dose-response curve to the right, but reduces the maximal amplitude of the responses [63]. Here we also checked the applicability of the calcium imaging with Case12 on the example of α1β1εδ nAChR inhibition by a well-known competitive antagonist, αBgt. A 5 min pre-incubation of Neuro2a cells with αBgt (100 nM) shifts the acetylcholine dose-response curve to the right, and the agonist EC_50_ value increases from 11.5 μM to 44.2 μM (S8 Fig). Moreover, we did not observe any reduction in the maximal amplitude of the cellular responses (S8 Fig). This indicates clearly that under conditions described in this article calcium imaging is applicable for the detection of competitive antagonism.

We compared BBIQA1, BBIQA2, and *d*-TC by this calcium imaging test and detected quite unusual effect of all three compounds on the acetylcholine dose-response curve for the α1β1εδ receptor: in addition to the curve shift to the right (with increasing agonist EC_50_ value, see Table 2), for all three compounds there was a concomitant reduction of peak acetylcholine responses (Fig 3C). This finding, along with the results of radioligand assay, supports our hypothesis that BBIQA1 and BBIQA2 inhibit adult muscle α1β1εδ nAChR by mixed mechanisms, in which both competitive and noncompetitive modes are involved. For *d*-TC this mixed mechanism was confirmed by the earlier data [60], where the same effect on the muscle-type nAChR was observed in the electrophysiology experiments.

In GH_4_C_1_ cells expressing α7 nAChR, *d*-TC competitively inhibits binding as previously reported [31]. BBIQA2 is also able to compete with [^125^I]-αBgt with a potency similar to *d*-TC, but BBIQA1 is considerably less potent. On the contrary, in calcium imaging experiments, we did not see any strong differences in the inhibitory potencies of all three compounds. Thus BBIQA1 might also interact with allosteric sites in α7 nAChR. A similar example has been reported for the muscle-type receptor [60], where the use of nondesensitizing agonist (e.g. DMPP) resulted in competitive inhibition by *d*-TC, instead of noncompetitive inhibition in the case of acetylcholine-evoked responses. Thus, the mechanism is dependent on the receptor activation mode [60]. However, we must be cautious, as the calcium imaging experiments were performed in the presence of positive allosteric modulator PNU120596 (see above), which decreases the receptor desensitization.

The data for α3β2, α4β2, and α9α10 nAChRs show that against them BBIQAs and *d*-TC have very similar inhibitory potencies. A comparable inhibitory activity of *d*-TC and BBIQAs against α9α10 nAChR is of considerable importance because these receptors are targets for the development of new analgesics, among which several α-conotoxins are being explored [64]. Both BBIQA1 and BBIQA2 demonstrate some decrease in their inhibiting potencies at GABA_A_R in comparison with *d*-TC in electrophysiological experiments, but there were no significant differences between them in competition with Alexa Fluor 546 αCtx, which can bind both to orthosteric and allosteric sites [40]. Thus, the situation with GABA_A_R might also indicate a more complicated inhibitory mechanism, in which binding to orthosteric and allosteric sites could be involved.

At 5-HT_3A_R, however, BBIQA1 and BBIQA2 behave quite differently, being 5- and 87-fold less active than *d*-TC, thus demonstrating that compared to *d*-TC BBIQAs are less selective for 5-HT_3A_R than nAChRs. This finding may be important since some α7 nAChR targeting drugs are problematic because they also act on 5-HT_3_R. Further investigating of key structural elements responsible for mechanisms of action of these compounds might be very useful for future drug design.

## Supporting information

S1 Table Predicted affinity of the alkaloids from Matis Dart Poison and *d*-TC for AChBP from *A*. *californica*. (DOCX)

S1 Fig Computer docking of the BBIQA1 and BBIQA2 to *A*. *californica* AChBP. Docked structures overlay of (**a**) BBIQA1 (*purple*) and *d*-TC (*black*) revealing differences in the predicted binding modes and (**b**) BBIQA2 (*purple*) and *d*-TC (*black*) revealing similarity in the predicted binding modes. Despite the difference in binding configurations, both BBIQA1 (**c**) and BBIQA2 (**d**) were docked at the classic orthosteric site under the loop C of the AChBP.
(TIF)

S2 Fig Analytical HPLC data for BBIQA1 purification. (TIF)

S3 Fig MS data for the BBIQA1. (TIF)

S4 Fig Analytical HPLC data for BBIQA2 purification. (TIF)

S5 Fig MS data for the BBIQA2. (TIF)

S6 Fig Analytical HPLC data for BBIQA3 purification. (TIF)

S7 Fig MS data for the BBIQA3. (TIF)

S8 Fig Dose-response curves of acetylcholine (ACh)-evoked [Ca^2+^]_i_ rise in the absence (*grey circles*, EC_50_ = 11.5 ± 1.43 μM) and presence of 100 nM *α*Bgt (*violet circles*, EC_50_ = 44.2 ± 8.38 μM) in Neuro2a cells expressing mouse adult α1β1εδ nAChRs. Data are presented as mean ± SEM, n = 3.
(TIF)
