# Supplementary material for: Curare alkaloids from Matis Dart Poison: Comparison with d-tubocurarine in interactions with nicotinic, 5-HT3 serotonin and GABAA receptors
Source: PLoS One. 2019 Jan 4;14(1):e0210182. doi: 10.1371/journal.pone.0210182 (PMC6319706; doi:10.1371/journal.pone.0210182)
Supplement: S1 Table — (DOCX) [file pone.0210182.s001.docx]

**S1 Table. Predicted affinity of the alkaloids from Matis Dart Poison and *d*-TC for AChBP from *A. californica.***

| Compound | ***Molecular docking score, kcal/mol*** | | |
| --- | --- | --- | --- |
|  | **Mode 1** | **Mode 2** | **Mode 3** |
| ***d*-ТС** | **-10.09** | **-9.37** | **-9.56** |
| **BBIQA1** | **-10.22** | **-10.92** | **-12.76** |
| **BBIQA2** | **-9.44** | **-10.36** | **-9.58** |
