# Supplementary figures and images for: Curare alkaloids from Matis Dart Poison: Comparison with d-tubocurarine in interactions with nicotinic, 5-HT3 serotonin and GABAA receptors
Source: PLoS One. 2019 Jan 4;14(1):e0210182. doi: 10.1371/journal.pone.0210182 (PMC6319706; doi:10.1371/journal.pone.0210182)

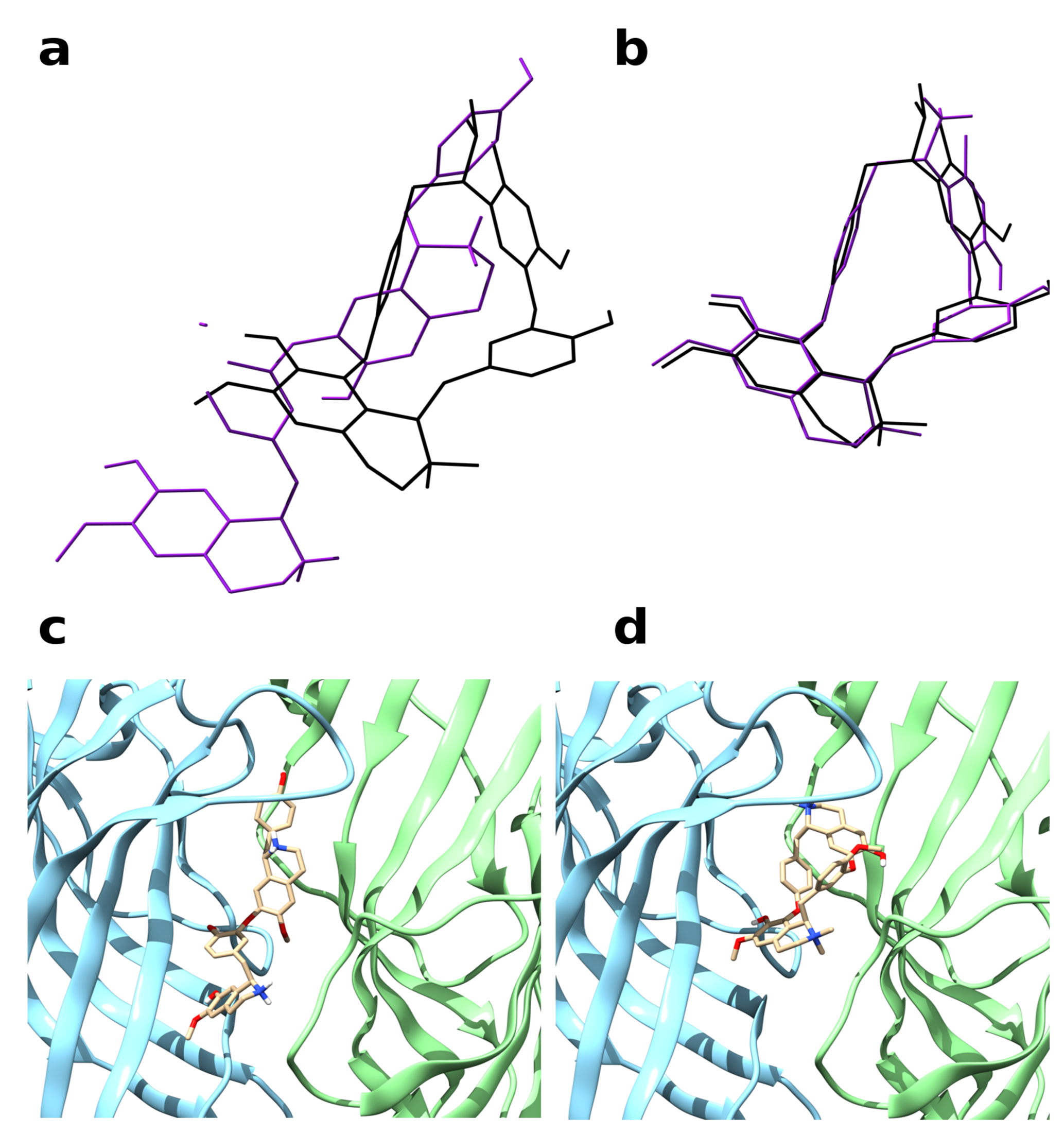

Supplement: S1 Fig — Docked structures overlay of (a) BBIQA1 (purple) and d-TC (black) revealing differences in the predicted binding modes and (b) BBIQA2 (purple) and d-TC (black) revealing similarity in the predicted binding modes. Despite the difference in binding configurations, both BBIQA1 (c) and BBIQA2 (d) were docked at the classic orthosteric site under the loop C of the AChBP. (TIF) [file pone.0210182.s002.tif]

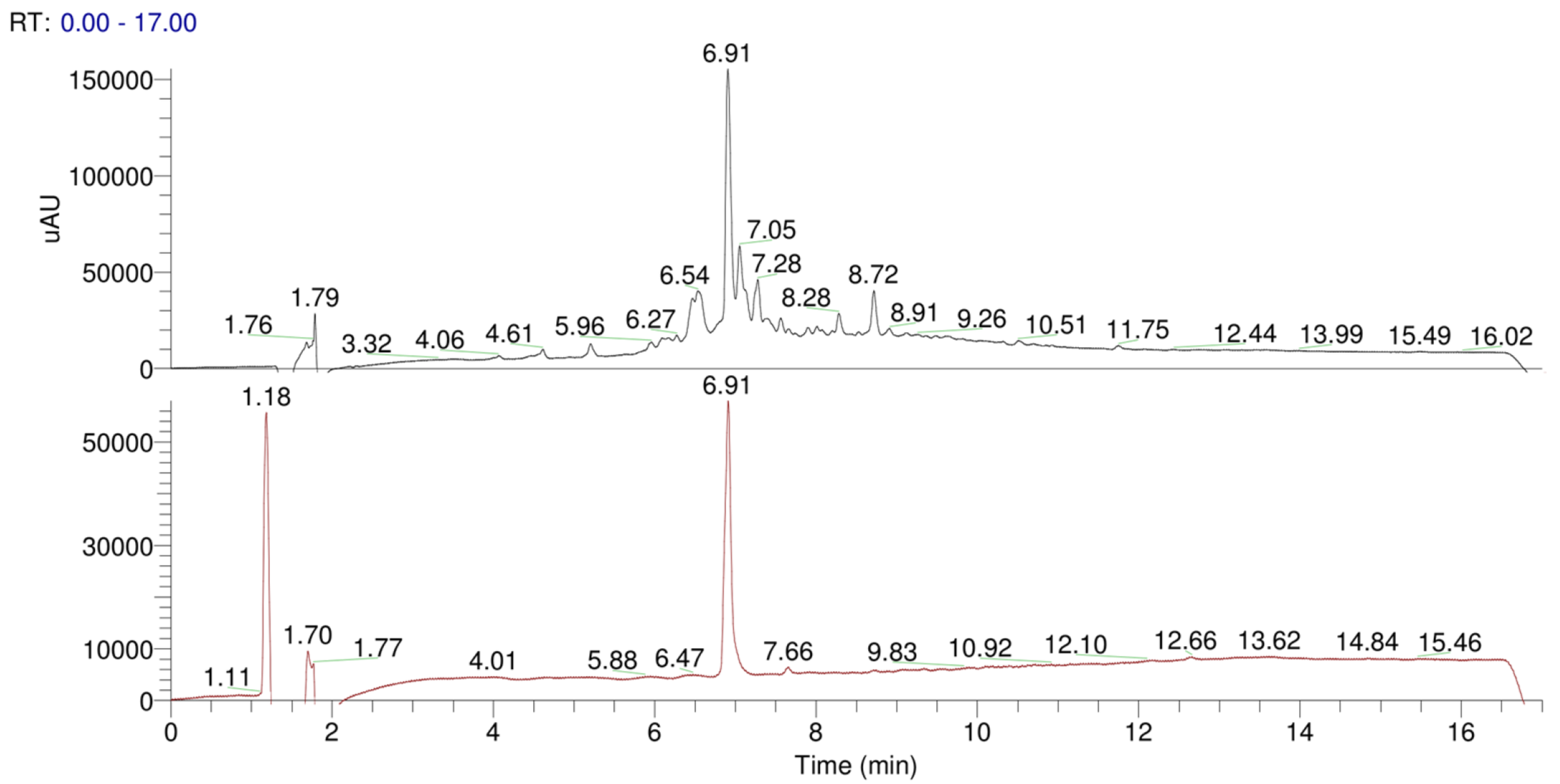

Supplement: S2 Fig — (TIF) [file pone.0210182.s003.tif]

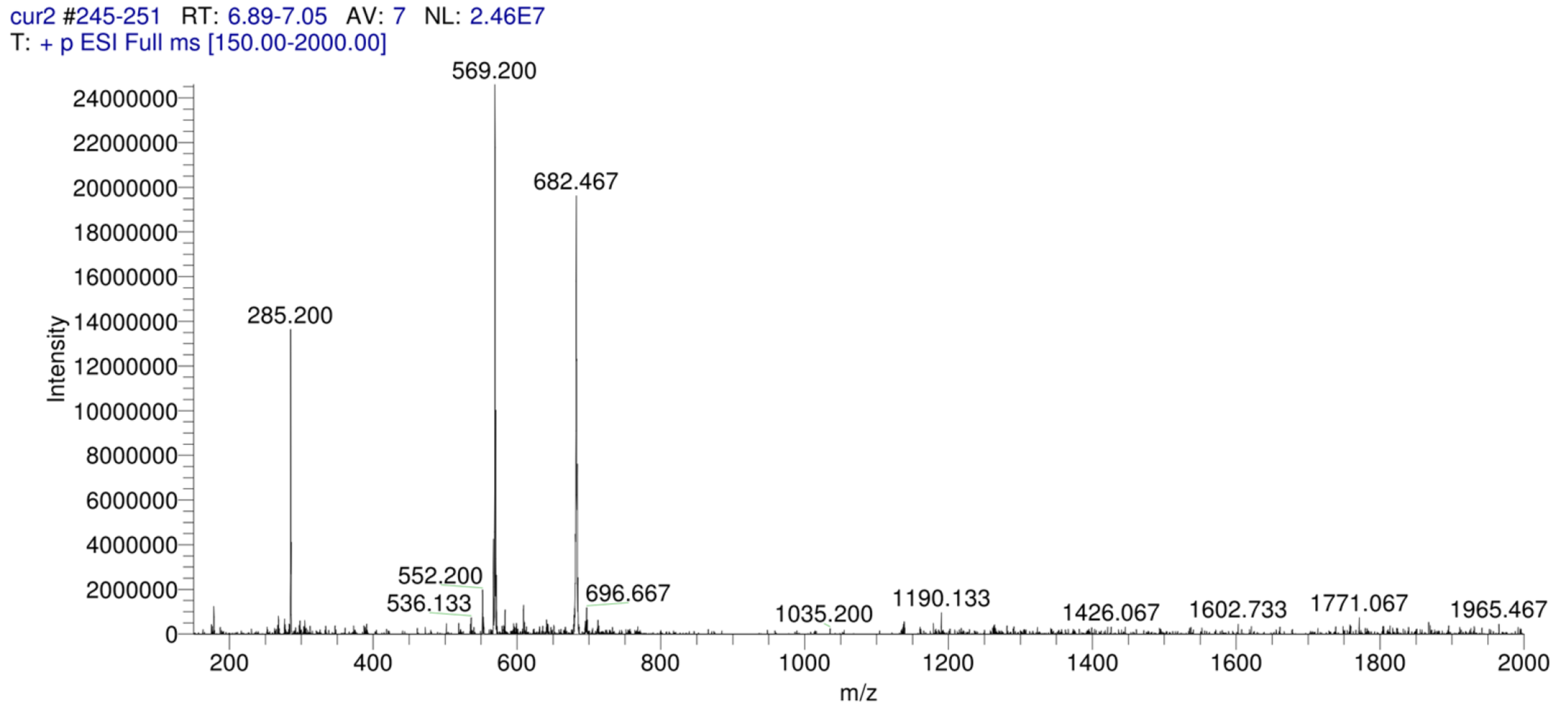

Supplement: S3 Fig — (TIF) [file pone.0210182.s004.tif]

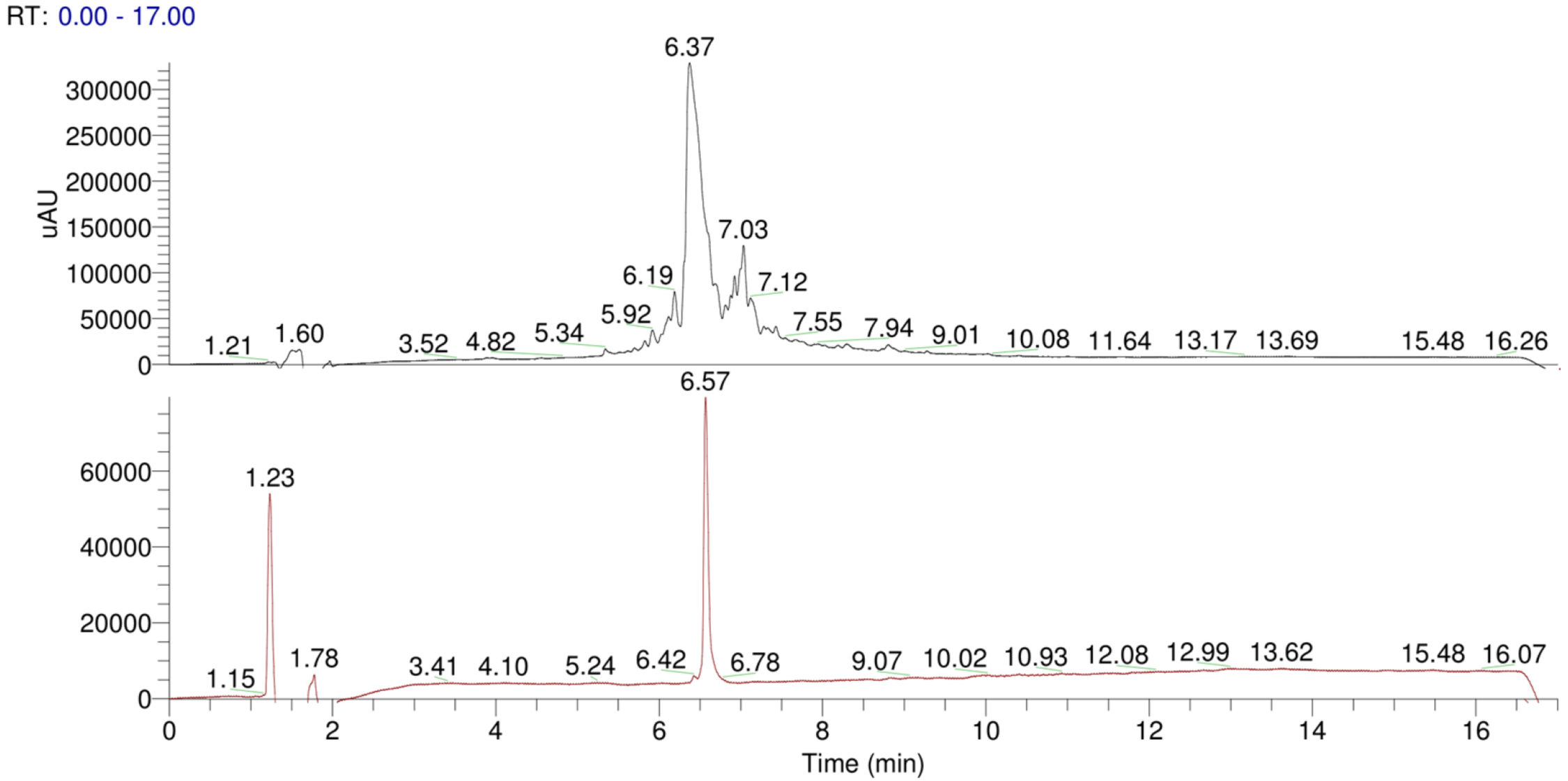

Supplement: S4 Fig — (TIF) [file pone.0210182.s005.tif]

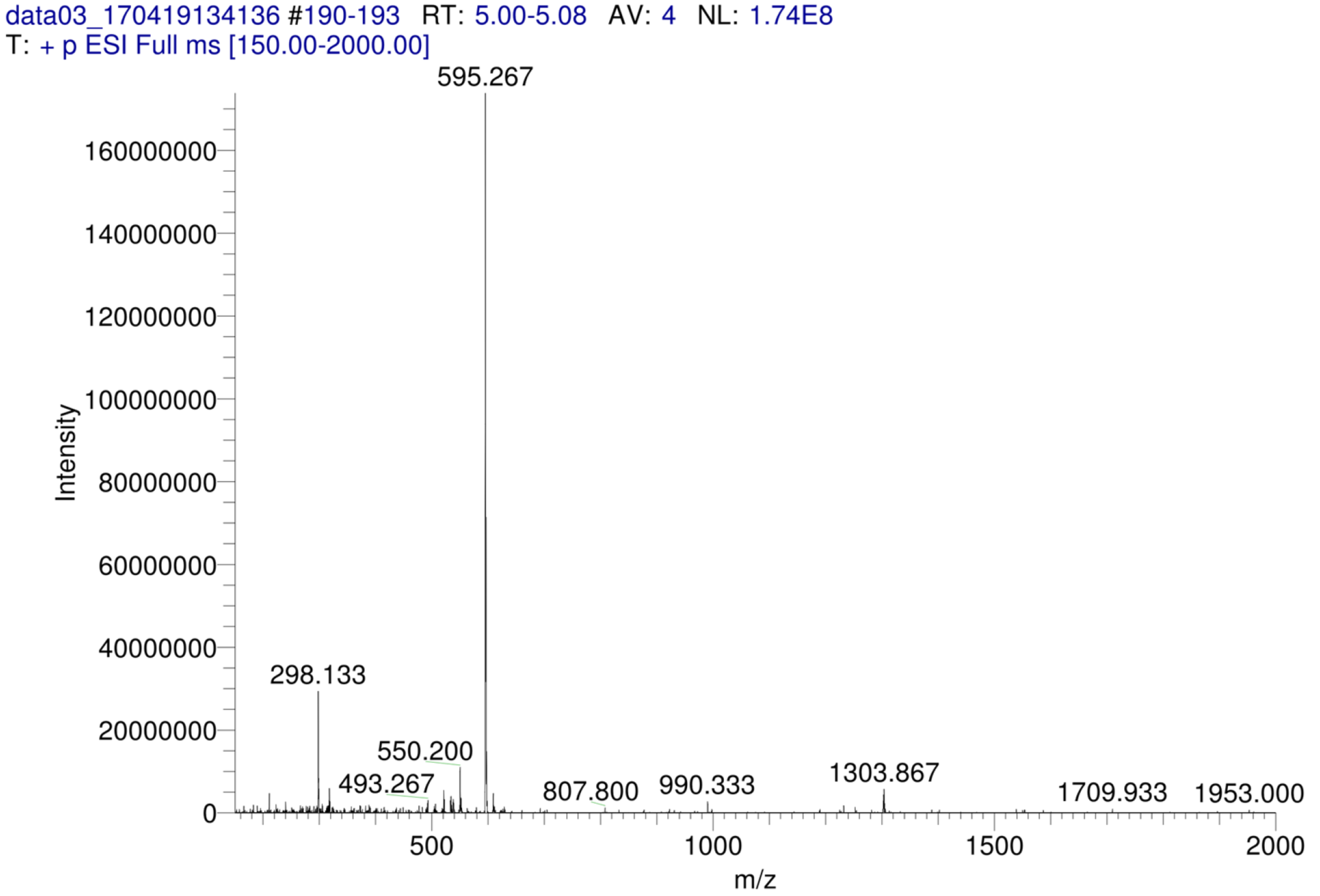

Supplement: S5 Fig — (TIF) [file pone.0210182.s006.tif]

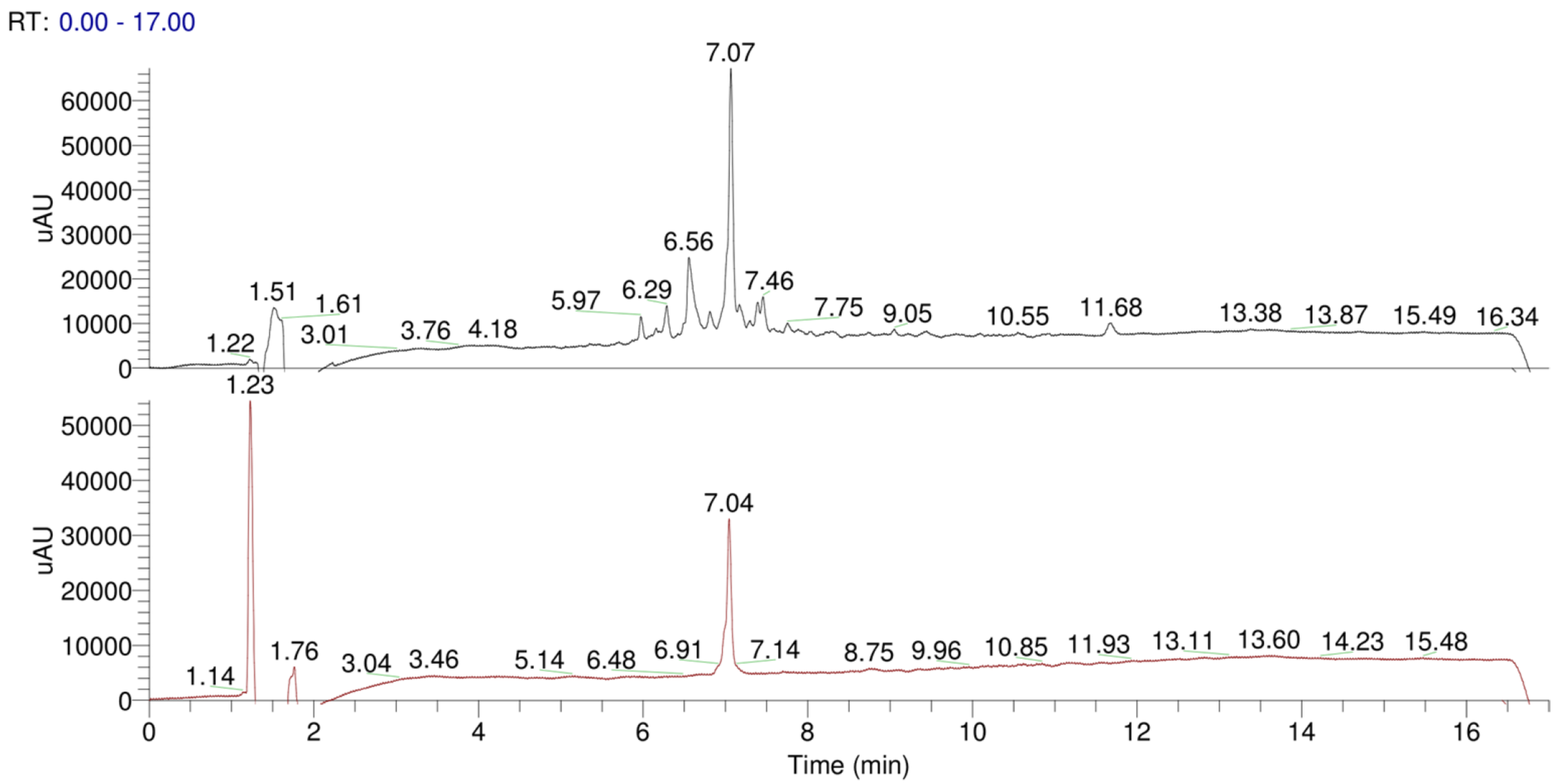

Supplement: S6 Fig — (TIF) [file pone.0210182.s007.tif]

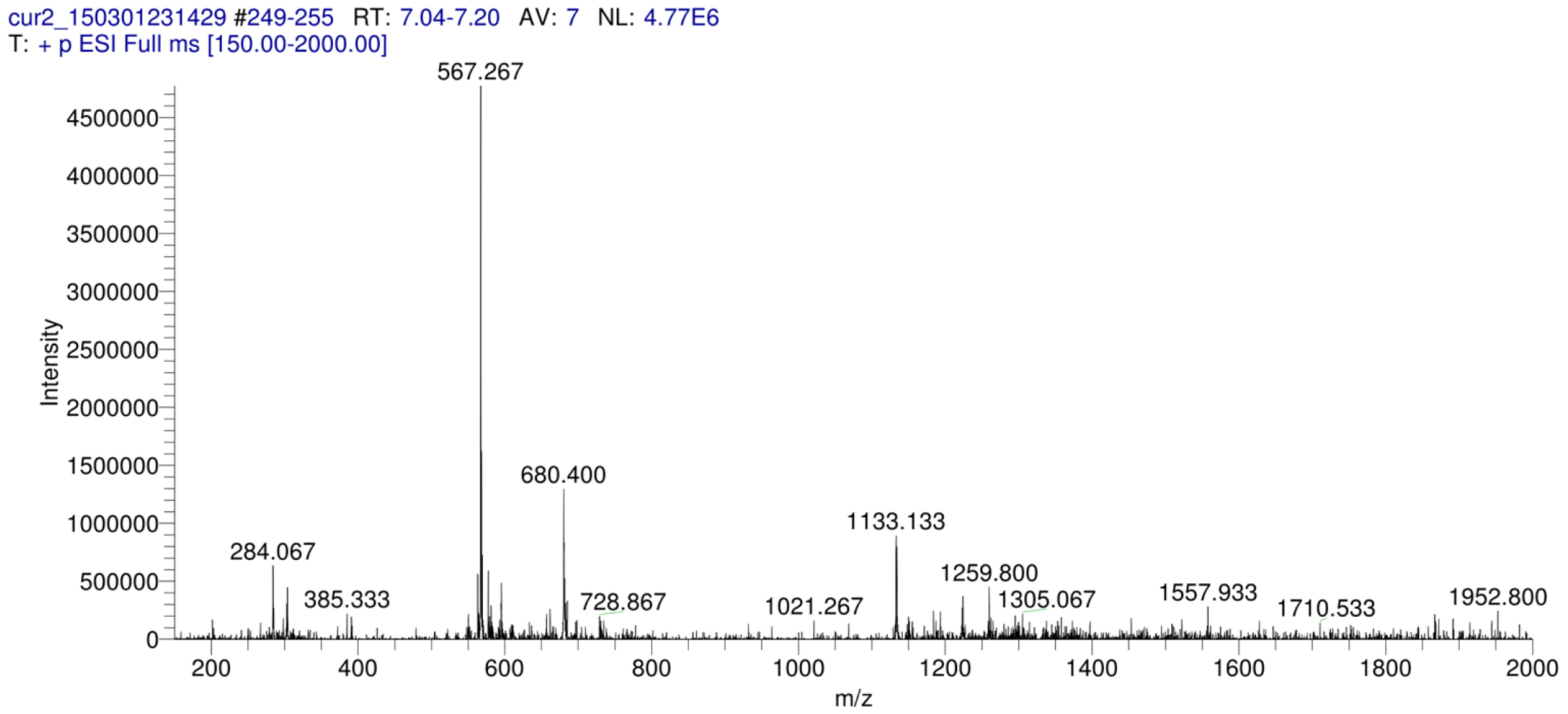

Supplement: S7 Fig — (TIF) [file pone.0210182.s008.tif]

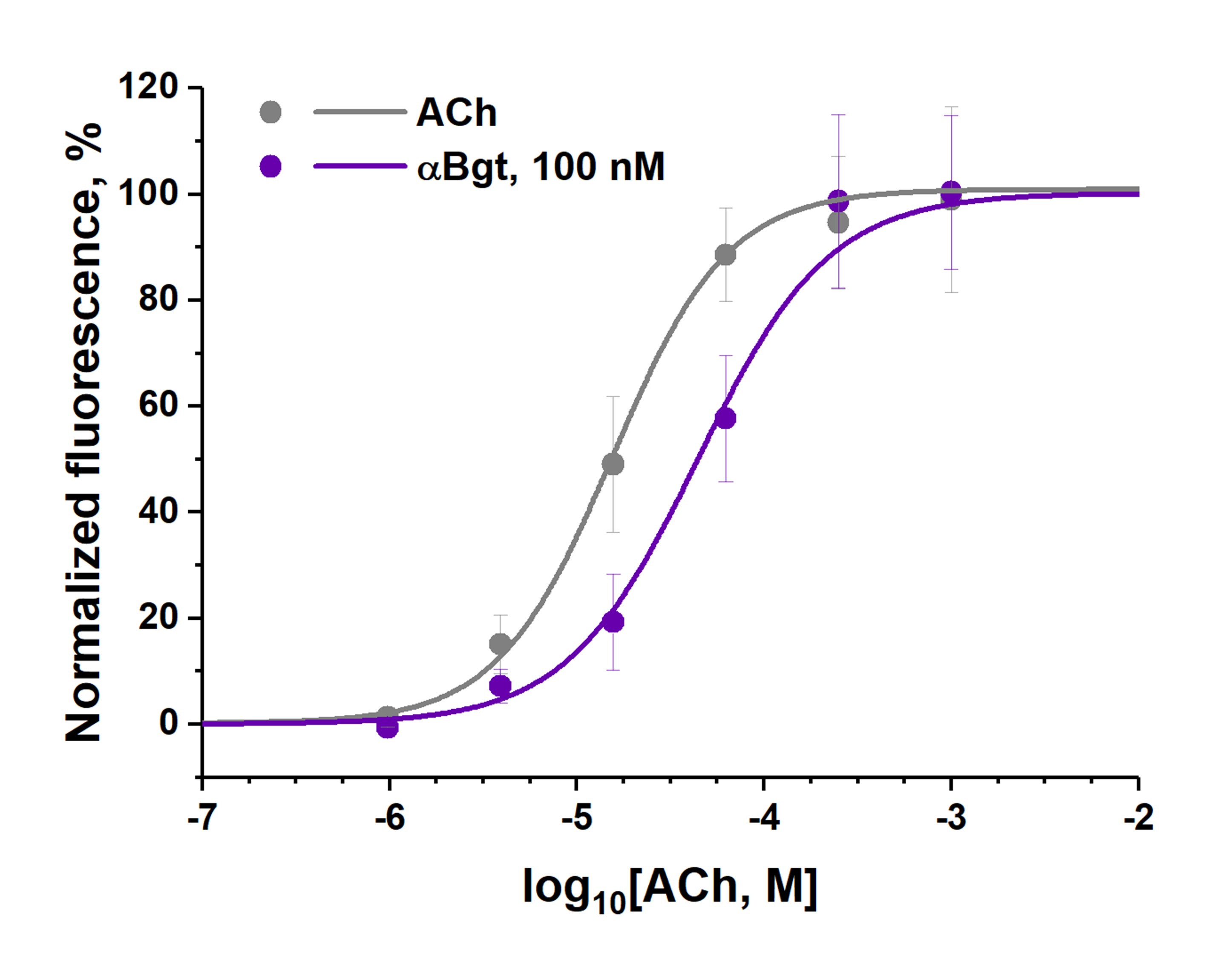

Supplement: S8 Fig — Data are presented as mean ± SEM, n = 3. (TIF) [file pone.0210182.s009.tif]
